# Complications associated to wound drainages in tumor spine surgery: a multicenter surveillance study from the German Spine Registry (DWG-Register)

**DOI:** 10.1038/s41598-022-23579-x

**Published:** 2022-11-21

**Authors:** Sebastian G. Walter, Maximilian Lenz, Christopher Gaisendrees, Georg Schlachtenberger, Krishnan Sircar, Peter Knöll, DWG Registry Study Group, Jan Siewe, Christopher Brenke, Yorck Rommelspacher, Ehab Shiban, Simon Bayerl, Christoph Mehren, Juan Manuel Vinas-Rios, Kourosh Zarghooni

**Affiliations:** 1 Department of Orthopedic Surgery and Traumatology, University Hospital Cologne, Joseph-Stelzmann-Str. 24, 50931 Cologne, Germany; 2 Department of Cardiothoracic Surgery, University Hospital Cologne, Cologne, Germany; 3 Department of Orthopedic Surgery and Traumatology, Leverkusen Hospital, Leverkusen, Germany; 4 Department of Neurosurgery, Bergmannsheil Buer Clinic, Gelsenkirchen, Germany; 5 Department of Spine Surgery, Severinsklösterchen, Cologne, Germany; 6 Department of Neurosurgery, University Hospital Augsburg, Augsburg, Germany; 7 Department of Neurosurgery, University Hospital Charite, Berlin, Germany; 8 Department of Spine Surgery, Schön Clinics, Munich, Germany; 9 Department of Spinal Surgery, Sanaklinik Offenbach Am Main, Offenbach, Germany

**Keywords:** Medical research, Oncology, Risk factors

## Abstract

There is an ongoing debate whether a surgical drainage is beneficial to prevent local accumulation of hematoma and to reduce the rate of wound infections, and neurological deficits. Data from the German Spine Society (DWG) registry were filtered for surgically treated spine tumor cases between 2017 and 2021. Cases were categorized into with (Group I) and without (Group II) placement of a surgical drainage. Subgroups were compared for demographic data, type of surgery, experience of the surgeon and postoperative surgical complications. 10,029 cases were included into final analysis (Group I: 3007; Group II: 7022). There was no significant difference between both groups regarding age or gender distribution. Average morbidity of patients was significantly elevated in Group I (p < 0.05) and the rates of invasive surgery were significantly increased in this group (p < 0.001). Overall complication rates were reported with 12.0% (Group I) and 8.5% (Group II). There were significantly more epidural hematoma (p < 0.001) and motor dysfunction (p = 0.049) as well as deep wound infections (p < 0.001) and implant failures (p = 0.02) in Group I. A surgical wound drainage cannot prevent epidural hematoma.

## Introduction

Surgical treatment of spine tumors is an important, growing but interdisciplinary challenging field of orthopedic surgery and neurosurgery. There is an ongoing debate whether a surgical drainage is beneficial to prevent local accumulation of hematoma and to reduce the rate of wound infections, and neurological deficits^1,2^. There is only limited evidence whether patients profit from wound drainage after spine tumor surgeries and if the postoperative complication rate is lower if a drainage is placed.

In theory, placement of a surgical drainage is preventive for development of postoperative hematoma and associated spinal cord compression, as the blood will not remain and clot in-situ^3^. Most commonly a redon drain is placed. If the postoperative course is uncomplicated and the volume of drainage is low, it can be removed after 24–48 h. The redon drain can be placed with or without suction using a sterile filter.

Multiple studies have been conducted to evaluate the benefits of placing a wound drainage. However, no data is available investigating the role of a wound drain in extradural tumor spine surgery^4^.

Therefore, this registry study was conducted to evaluate on a large, multicenter cohort of patients undergoing tumor spine surgery whether placement of a wound drainage is beneficial and associated to a reduced rate of peri-operative, surgical complications. The German Spine Society (DWG) initiated a registry that allows to collect data for perioperative parameters associated to spinal surgery and additional forms are available to optionally contribute patient related outcome measures.

## Material and methods

To receive data from the DWG-registry a study outline was sent to the German Spine Society. A positive ethical vote was received previously for contributing clinical data to the registry and to analyze registry data for scientific purposes. Data gathered through the V2-questionnaire form, which was introduced in 2017 were analyzed for this study. There were 230,240 data case-files for spinal surgeries registered between January 2017 and February 2022. Data were received without direct personal identifiers. Duplicates and non-tumor related cases were removed for final analysis. Cases without the answer eight (tumor) for the category “main pathology” were excluded from analysis.

For analysis V2-questionnaire items such as year of birth, gender, tumor type and dignity, tumor localization, reason for repeat surgery, most severely affected segment/vertebral body, extension of lesion, number of previous spine surgeries, BMI, therapeutic goal, morbidity, technology, number of blood transfusions, operation time, surgical decompression technique, fusion material, other surgical measures, extent of surgery, and postoperative complications were included for final data analysis.

Statistical analysis was carried out with SPSS statistics 25 for Windows (SPSS, Inc, an IBM company, Chicago, IL, USA). Descriptive statistics, including arithmetic mean value and standard deviation were calculated. Data are given as means ± standard deviation (SD) and ranges, if not indicated otherwise. The object of this study was to investigate whether placement of a wound drainage would result in less complications compared non-placement of a drainage.

For comparison of cases with and without wound drainage analysis of variances (ANOVA) was performed to detect differences between the groups and p-values < 0.05 were regarded as significant. Multivariate analysis was performed in case of univariate significance. Data groups were tested for homogeneity using the Chi-Square Test. All methods were performed in accordance with the relevant guidelines and regulations described by the policies of the Nature Portfolio journals.

## Results

### Demographic results

**Figure 1:**
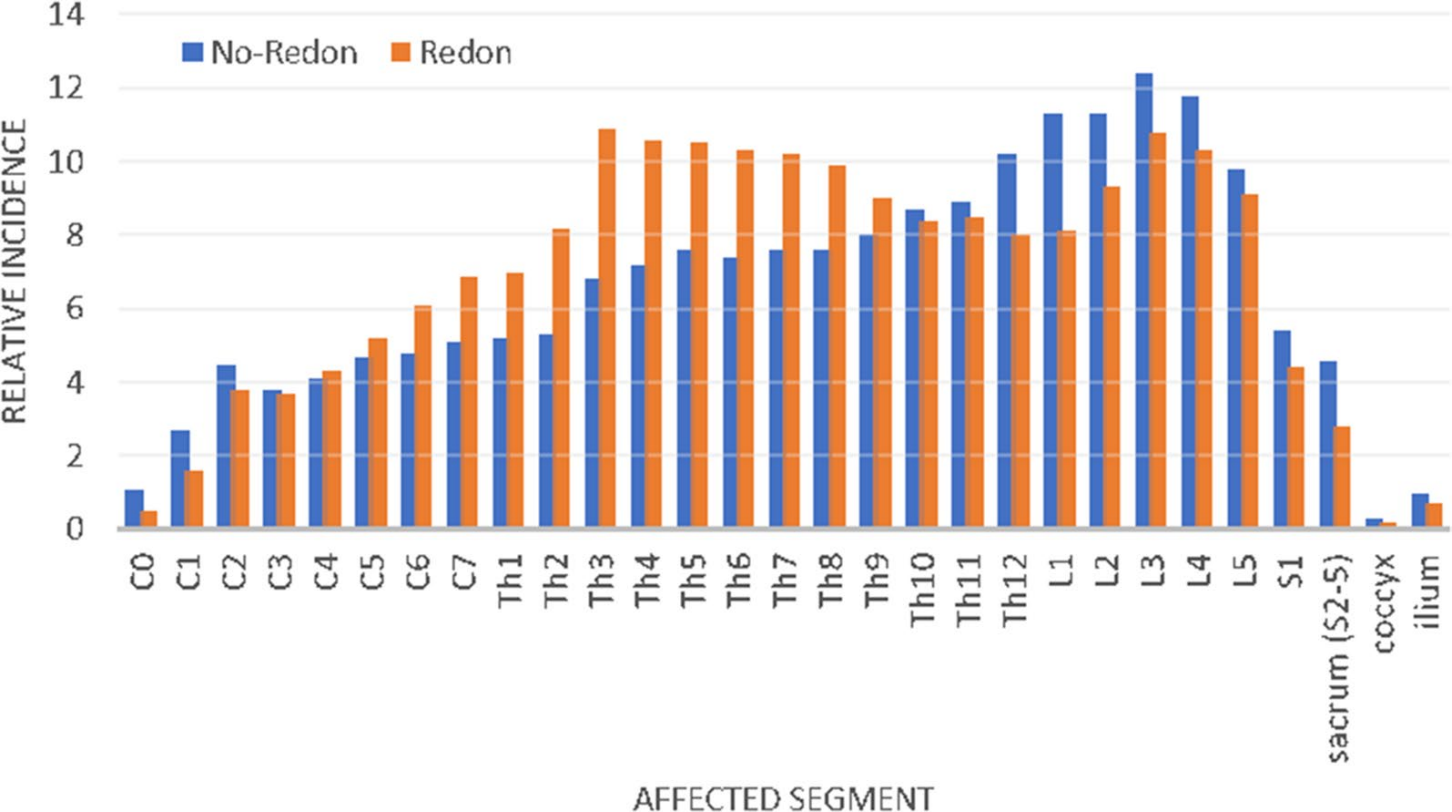
Graph depicting the relative incidences of affected vertebral segment that were treated surgically with (orange) and without (blue) surgical drain. Note that there were significant differences for segments C6-Th8 and Th12-L4 (p < 0.05) and in conclusion significantly more redons were placed after surgery at the upper spine, while significantly less redons were placed at the lumbar spine.

After filtering all registered cases for the main pathology “tumor” and removal of double registered cases, there were 10,029 cases for final analysis. 3007 cases received a wound drainage (Group I) during the surgical procedure, while the remaining 7022 cases (Group II) did not. There was no significant difference between both groups regarding demographic factors such as age or gender (Table 1). A significantly (p < 0.001) larger proportion of Group I had an ASA 4 score compared to Group II, while there were no significant differences for the other ASA categories. There were significant differences between both groups regarding the tumor type. Such, Group I contained 2251 (74.9%) malignant tumors, whereas there were 4208 (59.9%) malignant tumors in Group II (p < 0.001). In analogy, there were significantly (p < 0.001) more benign tumors in Group II (n = 1909 (27.1%)) compared to Group I (n = 565 (18.8%)). Tumor-like-lesions and tumors classified as “other” accounted for the remaining cases (Group I: n = 191 (6.4%) vs. Group II: n = 738 (10.5%)). The relative incidence of affected and thus surgically treated vertebral segments differed significantly (p < 0.05). Such, there was a higher rate of thoracic segments treated with redon drains and a higher rate of lumbar segments treated without a surgical drainage (Fig. 1).

**Table 1:** Overview of demographic data of patients that received a redon drainage and patients that did not for spinal tumor surgery. Age is expressed as the median with range. For the ASA score and surgeon credentials absolute number of cases is presented. Surgeon credentials: *SS* specialized spine, *BCO* board certified orthopaedic, *BCN* board certified neurosurgeon, *OiT* orthopaedic in training, *NiT* neurosurgeon in training. *p < 0.05.

	Redon	No redon	p-value
Total number of cases	3.007	7.022	
Male/female	1.731/1.276	3.807/3.215	0.371
Age	62.5 ± 15.5	61.2 ± 16.4	0.532
**ASA score**			
1	256 (8.5%)	747 (10.6%)	0.021*
2	946 (31.5%)	2.455 (35.0%)	
3	1.493 (49.7%)	2.848 (40.6%)	
4	106 (3.5%)	134 (1.9%)	
5	5 (0.2%)	6 (0.1%)	
Unknown	201 (6.7%)	832 (11.8%)	
**Credentials surgeon**			
SS	454 (41.5%)	899 (41.8%)	0.422
BCO	165 (15.1%)	261 (12.1%)	
BCN	396 (36.2%)	746 (34.7%)	
OiT	23 (2.1%)	107 (5.0%)	
NiT	50 (4.6%)	116 (5.4%)	
Other	5 (0.5%)	23 (1.1%)	
Not reported	1914 (63.6%)	4870 (69.4%)

### Surgical parameters

**Figure 2:**
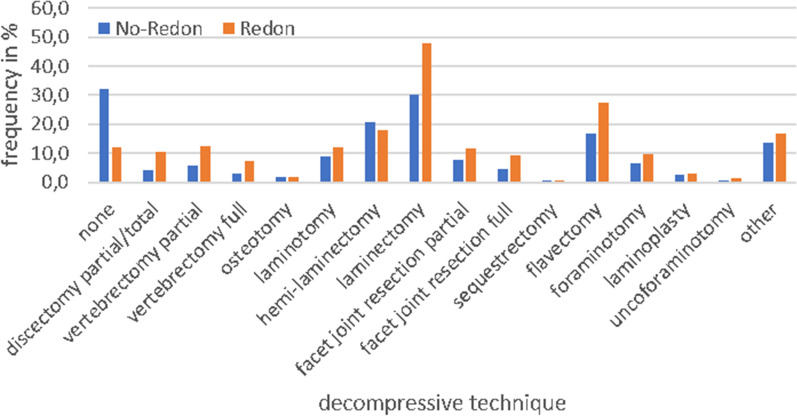
Relative frequency of decompressive surgical techniques for treatment of tumor spine cases regarding the placement of a surgical drain. The DWG V2 questionnaire allows for multiple choice of the decompressive surgical technique (e.g. According to the data set, there were 80 sequestrectomies and all of them were performed concomitantly with other decompressive measures, although this may appear questionable in the context of tumor spine surgery).

Surgery for diagnostic purposes (e.g. transpedicular biopsy) was significantly (p < 0.001) more often performed within the no-redon group. A significantly (p < 0.001) higher rate of dorsal instrumentation for stabilization was observed in Group I. The rate of uncemented pedicle screws was significantly higher in Group I (n = 1310 (43.6%)) compared to Group II (n = 1907 (27.2%)) as well as the rate of cemented pedicles screws (Group I: n = 281 (9.3%) vs. Group II: n = 490 (7.0%); p < 0.001). In analogy, significantly more cages were placed within Group I for stabilization (redon: 13.1% vs. no-redon: 4.9%; p < 0.001). Overall, the relative invasiveness of surgical treatment was higher within the redon group, as significantly more laminectomy (p < 0.001) and vertebrectomy (p < 0.001) procedures were performed (Fig. 2, Table 2).

**Table 2:** Decompressive surgical techniques used within the redon and the no-redon group. There were significant differences in the rates of surgical measures between groups and the redon group accounted for more invasive procedures. Significant values are in bold.

Decompressive technique	Total cohort n = 10,043	No-redon n = 7036	Redon n = 3007	p-value univariate	p-value multivariate	Odds ratio	Confidence interval
None	26.2	32.2	12.1	**< 0.001**	**< 0.001**	2.27	1.91–2.70
Discectomy partial/total	6.0	4.1	10.6	**< 0.001**	**< 0.001**	0.63	0.51–0.76
Vertebrectomy partial	7.7	5.6	12.6	**< 0.001**	**< 0.001**	0.60	0.50–0.71
Vertebrectomy full	4.2	2.9	7.3	**< 0.001**	**< 0.001**	0.50	0.39–0.63
Osteotomy	1.7	1.7	1.7	0.99			
Laminotomy	9.8	8.9	12.0	**< 0.001**	0.06	0.86	0.73–1.0
Hemi-laminectomy	19.9	20.8	17.9	**< 0.001**	**0.003**	1.25	1.08–1.44
Laminectomy	35.4	30.1	47.9	**< 0.001**	**< 0.001**	0.68	0.59–0.76
Facet joint resection partial	8.8	7.6	11.6	**< 0.001**	0.19	0.89	0.77–0.16
Facet joint resection full	5.9	4.4	9.4	**< 0.001**	0.06	0.84	0.69–1.0
Sequestrectomy	0.8	0.8	0.8	0.98			
Flavectomy	19.9	16.6	27.6	**< 0.001**	**< 0.001**	0.73	0.65–0.83
Foraminotomy	7.5	6.6	9.5	**< 0.001**	0.81	0.91	0.86–1.11
Laminoplasty	2.8	2.7	3.0	0.34			
Uncoforaminotomy	0.8	0.5	1.4	**< 0.001**	0.39	0.61	0.64–1.1
Other	14.8	13.8	16.9	**< 0.001**	0.71	0.97	0.86–1.1

In both groups the majority of cases showed an extent of the surgically treated lesion of one vertebral segment only (Group I: n = 1426 (47.4%) vs. Group II: n = 3844 (54.7%)). Only a minor percentage of cases showed an extent of the tumorous lesion of four or more vertebral segments in both groups (Group I: n = 403 (13.4%) vs. Group II: n = 1657 (23.6%; p < 0.001). In the majority of cases duration of surgery was less than 3 h (Group I: 62% respective Group II: 76%). The majority of cases in both groups was operated by a posterior approach (Group I: n = 2757 (92%) vs. Group II: n = 6590 (94%); p = 0.182). There were significantly (p < 0.001) more blood transfusions with more than two units of packed red blood cells in Group I (n = 138 (4.6%)) compared to Group II (n = 121 (1.7%)). While the number of cases receiving a cell saver therapy was limited to n = 6 (0.2%, Group I) respectively n = 7 (0.1%, Group II) and did not differ significantly between both groups (p = 0.056), the vast majority of cases did not receive blood transfusions (Group I: n = 803 (26.7%); Group II: n = 1891 (26.9%)).

### Surgical complications

There were significant differences between both groups regarding postoperative complications that might be contributed to the placement of a redon drain (Table 3). Noteworthy, the rate of epidural hematoma, implant failure and deep wound infections was significantly higher for Group I after multivariate testing. There were 148 different centers that contributed to the analyzed data set. There were no center-specific complications.

**Table 3:** Multivariate analysis of surgical complications Redon vs. No Redon. p-values < 0.05 were considered statistically significant. For multivariate analysis risk factors such as BMI, drainage, ASA, age, type of surgical technique, experience of the surgeon were included. Significant values are in bold.

Surgical complications	Total cohort n = 10,043	No-redon n = 7036	Redon n = 3007	p-value	p-value multivariate	Odds ratio	Confidence interval
None	90.3	91.5	88.0	**< 0.001**	0.76	0.95	0.67–1.31
Epidural hematoma	1.2	1.0	1.9	**< 0.001**	**0.002**	0.52	0.36–0.79
Other hematoma	0.7	0.6	1.0	0.07			
Radiculopathy	0.3	0.3	0.4	0.32			
CSF leak/pseudomeningocele	1.1	1.1	1.1	0.99			
Motor dysfunction	2.5	2.3	3.0	**0.05**	0.06	0.69	0.48–1.01
Sensory dysfunction	1.9	2.0	1.8	0.31			
Bowel/bladder dysfunction	0.7	0.8	0.7	0.65			
Wound infection superficial	1.1	0.9	1.7	**< 0.001**	0.73	0.66	0.42–1.04
Wound infection deep	1.1	0.8	1.9	**< 0.001**	**< 0.001**	0.45	0.29–0.71
Implant malposition	0.5	0.5	0.8	**0.05**	0.26	0.70	0.38–1,29
Implant failure	0.2	0.1	0.4	**0.003**	**0.02**	0.33	0.13–0.84
Wrong level	0	0.0	0.01	0.21			
Recurrent nerve paresis	0.1	0.1	0.2	0.08			
Other	0.1	0.0	0.2	**0.03**	**0.03**	0.16	0.03–0.83
Not documented	1.5	1.3	2.1	**0.001**	**0.01**	0.58	0.38–0.89

Major therapeutic goals were axial (redon: 67.5% vs. no redon: 51.8%) and peripheral pain relief (redon: 37.3% vs. no-redon: 28.9%) for both groups, followed by functional/motor/sensory improvement (redon: 42.6%, 42.4%, 38.1% vs. no-redon: 32.4%, 31.5%, 30.4%).

## Discussion

Data of the German Spine Registry display a large series of spine surgeries performed in Germany during the past five years and numbers are approximative for spine surgery in tumor patients.

To the best of our knowledge, this is the first registry study investigating the benefits of surgical drain in tumor spine surgery.

Evidence for usage of wound drainages is limited and the application is rather generous. Placement of a surgical drainage can be justified by assumed prevention of hematoma and less spinal cord compression with neurological comprise, which has not been proven by the presented data. In theory, surgical drain may be associated with a higher rate of deep wound infections due to the direct connection with the outside environment, which is supported by the presented data showing significantly higher deep wound infection rates. The risk of developing deep wound infection after placement of surgical drain seems to be time-dependent. Usually wound drainage is claimed to be removed within 24–48 h after surgery, however, time threshold until occurrence of a surgical site infection remains unclear^5^. So far, there is no evidence and consensus that placement or non-placement of a surgical drain would result in higher rates of revision surgery. One study suggests that patients which received a surgical drain after spinal surgery had a prolonged hospital stay length^6^. However, this was not reproduced by other cohorts and all of these studies did not treat tumor patients^7,8^.

At first glance, data of the current study may seem conflicting with the aforementioned theory of less neurologic comprise after placement of a wound drainage. In the analyzed cohort, rates for postoperative complications such as epidural hematoma, motor dysfunction, deep wound infections and implant failure were significantly higher if patients had received a wound drainage (Table 2). However, there were significantly more invasive surgeries performed within this group compared to the *no-redon* cohort (Fig. 2). The latter might explain higher epidural bleeding rates. The rate of diagnostic goals for surgery was significantly smaller within the *redon* group, which might indicate a relatively smaller percentage of biopsy- and less invasive procedures. Furthermore, this cohort showed a significantly higher morbidity rate as expressed by the ASA-score compared to the *no-redon* cohort. An overall inferior health status within the *redon* group is likely to correlate with a negative affection of the immune system. This could explain significantly higher rates of deep wound infections that might subsequently result in higher rates of implant failure. Considering these factors of a more vulnerable cohort of patients receiving a wound drainage, might explain for significantly elevated complication rates within this group. It remains questionable whether complication rates would have been even higher for this collective if no wound drainage had been placed. Therefore, the placement of a wound drainage should be considered carefully until further evidence will advocate its placement or non-placement.

Although this study included patients from multiple centers and although the reporting according to the V2 questionnaire is highly standardized, there are limitations. The German Spine Society (DWG) Registry is not monitored and it has been shown before, that data within this registry may be biased^9^. Such, the number of primary malignant (n = 3026) tumors was reported to be as high as those of secondary malignant (n = 3600), which is highly unlikely. The DWG registry does not require documentation of the final histopathological diagnosis, which might solve this problem. Furthermore, underreporting of complications has been observed previously^10^ and complication rates of 12.0% (redon) respective 8.5% (no redon) seem to be unexpectedly low within this vulnerable collective of tumor patients^11^.

## Conclusions

In conclusion, a surgical drain cannot prevent epidural hematoma in tumor spine surgery but is also associated with an increased risk of deep wound infections and implant failure within a vulnerable cohort. This current study adds further evidence to the ongoing debate whether placement of a wound drain is beneficial in spine surgery. Further prospective and randomized studies are needed to clarify this question ultimately.

## Data Availability

The data that support the findings of this study are available on request from the corresponding author. Original data can be received from the German Spine Society (DWG) registry upon request as well.
